# Ergonomic hand positioning overcomes visual perception mismatch in nonsimulated robotic colorectal surgery

**DOI:** 10.1093/jscr/rjae143

**Published:** 2024-03-15

**Authors:** Shing Wai Wong, Ranah Lim, Xiuling Jasmine Wong, Philip Crowe

**Affiliations:** Department of General Surgery, Prince of Wales Hospital, Sydney, New South Wales, 2031, Australia; Randwick Campus, School of Clinical Medicine, The University of New South Wales, Sydney, New South Wales, 2052, Australia; Department of General Surgery, Prince of Wales Hospital, Sydney, New South Wales, 2031, Australia; Department of General Surgery, Prince of Wales Hospital, Sydney, New South Wales, 2031, Australia; Department of General Surgery, Prince of Wales Hospital, Sydney, New South Wales, 2031, Australia; Randwick Campus, School of Clinical Medicine, The University of New South Wales, Sydney, New South Wales, 2052, Australia

**Keywords:** ergonomics, visual perception mismatch, clutch activation, robotic colorectal surgery

## Abstract

The aim of the study was to compare the internal instrument and external surgeon hand positions to determine whether visual perception mismatch (VPM) is a factor during robotic colorectal surgery. Continuous video footage of 24 consecutive robotic colorectal surgery cases were analysed concurrently with sagittal video recordings of surgeon hand positions. Separated sagittal hand positions would indicate nonergonomic positioning without clutching of the robotic controls, either matching the on-screen up/down instrument tip positions (no VPM) or in the opposite direction (true VPM). Variables (30-min surgery time blocks, anatomic target, and task performed), which resulted in hand separation or VPM, were analysed. Operating with the presence of VPM for more than one duration occurred 51 times and nonergonomic sagittal hand positioning occurred 22 times. For an experienced robotic surgeon, ergonomic positioning of the hands is favoured over adjustment for VPM despite the potential higher mental workload.

## Introduction

Improved visualization is a perceived advantage of robotic surgery [1]. There are visualization ergonomic disadvantages of the robotic system, mainly relating to mismatch and fatigue [2]. Cognitive workload may be increased for the robotic surgeon because of reduced situational awareness, communication difficulties, need to control more instruments, and lack of haptic feedback [3]. Robotic surgery can potentially introduce a visual perception mismatch (VPM) that can add an additional cognitive load [4]. VPM can occur with brain sensory misalignment of the positions of console surgeon hands and robotic instruments as visualized through the binoculars. Activation of the clutch temporarily disconnects the robotic arms guidance from the console so that the console controls can be repositioned to allow for additional movement [3]. The movement ratio of the robotic arms relative to the surgeon console controls is also dependent on the amount of motion scaling used and can add to this VPM. Motion scaling has been shown to improve task time, enhance accuracy, and reduce error rates [5, 6]. Studies have shown that use of the clutch control is more frequent with increasing robotic experience [7, 8].

Abiri *et al*. found significantly more peg drops and longer time to completion with a peg transfer task from one robotic instrument to another when novice surgeons were operating with a single-axis misalignment during simulation surgery [4]. Visual and proprioceptive position estimates may be different because of their independent processing, especially when there is spatial offset [9]. However, VPM may not be clinically relevant because the experienced surgeon can combine different unimodal sensory cues, especially from a common cause, to a single multisensory estimate which minimizes variance [9].

The aim of this clinical study was to evaluate situations when surgeon hands were not aligned side by side in the sagittal plane and/or when there was evidence of VPM during nonsimulated robotic colorectal surgery. Up–down separation of the hands at the robotic console can occur in the same (matched) or in the opposite (mismatched) directions with the on-screen instrument tip positions. Surgeon preference for better hand and upper limb ergonomics or avoidance of VPM was assessed.

## Materials and methods

Robotic surgery for colorectal disease were performed by a single colorectal surgeon (S.W.W.) with the da Vinci Xi™ system at the Prince of Wales Private Hospital. The right-handed surgeon has performed over 150 robotic colorectal surgery cases, with demonstrated proficiency past the learning phase [10]. The four robotic ports were placed obliquely in a straight line as recommended by the manufacturer. For all colonic resections, two left arms and one right arm configuration was the preferred set-up. In most cases, the left most robotic arm was used for retraction. The two robotic arms adjacent to the camera-holding robotic arm were the arms used primarily during surgery.

**Figure 1:**
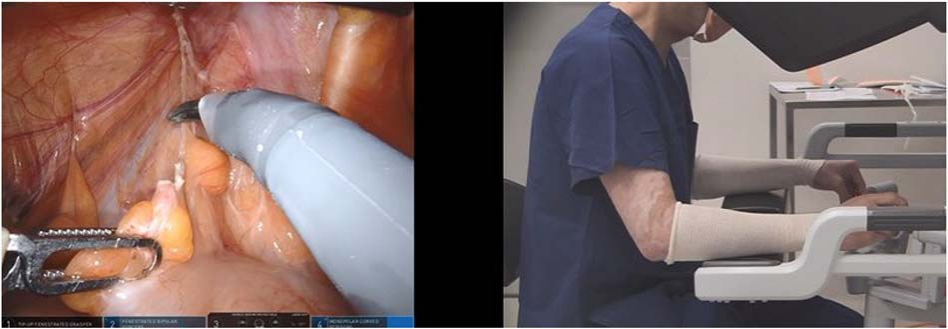
Ergonomic VPM; the instrument tip positions are misaligned in up–down position and the surgeon hands are aligned at the robotic console (scissors in right hand and bipolar forceps in left hand).

**Figure 2:**
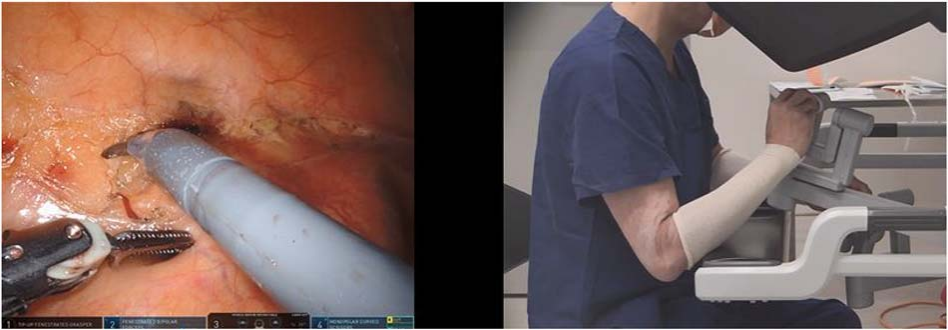
Nonergonomic and no VPM; the instrument tip positions are misaligned in up–down position and the surgeon hands match this misalignment at the robotic console (scissors in right hand and bipolar forceps in left hand).

**Figure 3:**
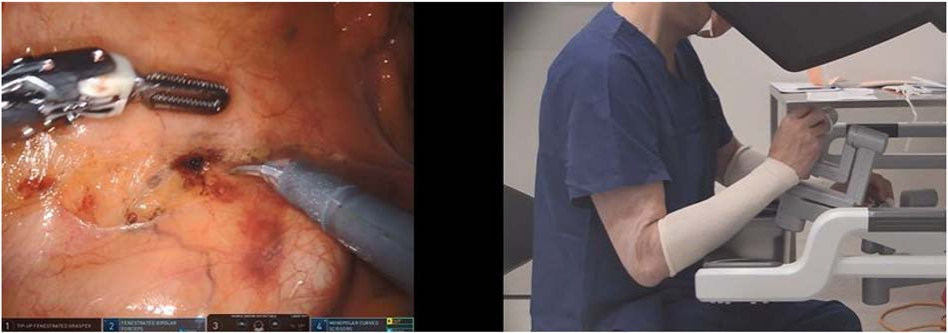
True VPM; the instrument tip positions are misaligned in up–down position and the surgeon hands are misaligned in the opposite direction at the robotic console (scissors in right hand and bipolar forceps in left hand).

Continuous video footage of 24 consecutive robotic colorectal surgery cases from September 2022 to April 2023 were analysed side by side with concurrent video recording of the lateral view (sagittal plane) of the surgeon sitting at the robot console (Figs 1–3). The video recording was performed with the robot and the operating theatre overhead light cameras. Ethics for this study were sought and granted by the South Eastern Sydney Local Health District HREC, reference number: 2021/ETH11587. The patients consented to participate in the study.

Two investigators independently analysed the videos. Their findings were compared, and any discrepancies were discussed until a consensus was reached. The position of the hands at the robot console (as recorded from a lateral perspective) were compared with the on-screen position of the tip of the instruments inside the abdominal cavity. The hands were aligned if the sagittal surgeon video revealed overlap of the hands (i.e. at the same level). This can occur when the instruments on-screen were not aligned in an up/down direction—which we have termed ‘ergonomic VPM’ (Fig. 1). Hand separation for >1-min duration was documented when there was distinct visualization of both hands without overlap at the robotic console, with regards to up–down positioning. Transient hand separation (<1 min) was common when there was transitioning to different parts of the operation, and this was not included in the analysis. Separated sagittal hand positions would indicate positions when the surgeon had not clutched in the robotic controls, either matching the on-screen up–down instrument positions (nonergonomic and no VPM) (Fig. 2) or in the opposite direction. The situation when the hand and instrument alignments were in the opposite direction has been termed as ‘true VPM’ for the purposes of the study (Fig. 3). The influence of variables on situations when the surgeon hands were not aligned in the sagittal plane or when VPM occurred was studied. The variables studied were time block of the surgery (divided into 30-min blocks), anatomic target, and task performed (dissection, preparing, stapling, or suturing).

## Results

The mean age of the patients was 63 (range: 22–87) (Table 1). There were 6 female patients and 18 male patients. Fourteen patients had robotic surgery for cancer, four for endoscopically unresectable polyps, four for diverticular disease, one for endometriosis, and one for inflammatory bowel disease. All patients had total robotic colorectal surgery without open conversion. Nine patients underwent right hemicolectomy, two left hemicolectomy, one Hartmann procedure, one reversal Hartmann procedure, four high anterior resection, five low anterior resection, one ultralow anterior resection, and one subtotal colectomy. The mean robotic console time was 215 min (range: 118–383 min).

**Table 1:** Patient demographics.

Patient number	Age	Gender	Total console time (min)	Surgery type	Indication	True VPM	Ergonomic VPM	No VPM and nonergonomic	Total either VPM or nonergonomic
1	69	M	175	LHC	C		6		6
2	69	F	285	HAR	C			1	1
3	87	M	234	LAR	P	1	2		3
4	79	M	273	H	DD		2	1	3
5	50	M	229	RHC	C		3		3
6	54	M	330	HAR	C		2		2
7	74	M	130	RHC	P	1	2	9	12
8	82	M	202	HAR	P		1	2	3
9	23	M	165	RHC	C		1		1
10	40	F	118	RHC	E		1		1
11	22	M	138	STC	IBD			1	1
12	71	M	154	RHC	C	1	4	1	6
13	72	F	140	RHC	C		1		1
14	77	M	154	LHC	C		1		1
15	59	F	316	LAR	DD		3		3
16	55	M	218	LAR	C		2		2
17	55	M	160	rH	DD		1		1
18	78	M	383	ULAR	C		2	1	3
19	62	M	213	RHC	C		1		1
20	73	M	277	RHC	C		2		2
21	65	M	191	HAR	C		1		1
22	62	F	244	LAR	P	2	5	1	8
23	75	F	220	LAR	DD		1		1
24	64	M	215	RHC	C		2		2
Total						5	46	17	68

LHC = left hemicolectomy; HAR = high anterior resection; LAR = low anterior resection; H = Hartmann procedure; RHC = right hemicolectomy; STC = subtotal colectomy; rH = reversal Hartmann; ULAR = ultralow anterior resection; C = cancer; P = polyp; DD = diverticular disease; E = endometriosis; IBD = inflammatory bowel disease.

The total number of times the surgeon’s hands were in ergonomic side-by-side positions, but the instrument tips were malaligned in up–down positions (ergonomic VPM) for >1-min duration was 46. The left-hand instrument tip (relative to the right-hand instrument tip) was down in 40 situations and up in 6 situations. The total number of times with nonergonomic hand positions which were matched by the instrument tips (nonergonomic and no VPM) for >1-min duration were 17. The right hand and right-hand instrument tip were up on seven occasions; the left hand and left-hand instrument tip were up on nine occasions; and both hands and instrument tips were up on one occasion. The nonergonomic hand position and no VPM combination occurred 14 times in two cases and was otherwise uncommon. There were five occasions when surgery continued despite the hands and the instrument tips being oriented in completely the opposite up–down directions (true VPM) for >1-min duration. This occurred twice with dissection of the hepatic flexure, twice with dissection of the inferior mesenteric artery, and once with dissection of the mesorectum.

Nonergonomic sagittal hand positioning for >1-min duration occurred 22 times (5 times with VPM). This occurred more frequently during later stages of the surgery (Graph S1). Operating with nonergonomic sagittal hand positioning for >1-min duration involved dissection in 13 situations, preparing in 5 situations, stapling in 2 situations, and suturing in 2 situations (Table 2). The target was ileocolonic anastomosis in five situations, rectum in four situations, hepatic flexure in four situations, colon mesentery in three situations, inferior mesenteric artery in three situations, transverse colon in two situations, and descending colon in one situation.

**Table 2:** Part of procedure performed resulting in either nonergonomic positioning or VPM for >1-min duration.

Patient number	Part of procedure performed
1	Mobilizing splenic flexure off lateral abdominal wall
	Mobilizing splenic flexure off spleen
	Mobilizing splenic flexure off Gerota’s fascia
	Mobilizing splenic flexure off spleen
	Mobilizing sigmoid colon off lateral abdominal wall
	Mobilizing sigmoid colon off lateral abdominal wall
2	Dissecting mesorectum
3	Mobilizing sigmoid colon off lateral abdominal wall
	Dissecting mesorectum
	Dissecting around inferior mesenteric artery
4	Dissecting caecal mesentery medially
	Mobilizing sigmoid colon off lateral abdominal wall
	Dissecting mesorectum
5	Mobilize ileocolic region laterally
	Dissecting ascending colon mesentery
	Dissecting ascending colon mesentery
6	Mobilizing sigmoid colon laterally
	Mobilizing descending colon laterally
7	Dissecting descending colon mesentery
	Mobilizing hepatic flexure
	Mobilizing hepatic flexure
	Mobilizing hepatic flexure
	Dissecting transverse colon mesentery
	Dissecting transverse colon mesentery
	Preparing transverse colon
	Stapling transverse colon
	Preparing small bowel and transverse colon for anastomosis
	Preparing small bowel and transverse colon for anastomosis
	Anastomosis suturing
	Anastomosis suturing
8	Mobilizing sigmoid colon off lateral abdominal wall
	Preparing inferior mesenteric artery pedicle
	Preparing small bowel and transverse colon for anastomosis
9	Mobilizing ascending colon laterally
10	Mobilizing ascending colon laterally
11	Dissecting descending colon mesentery
12	Taking omentum off transverse colon
	Taking omentum off transverse colon
	Taking omentum off transverse colon
	Taking omentum off hepatic flexure
	Taking omentum off hepatic flexure
	Mobilizing hepatic flexure
13	Mobilizing ascending colon laterally
14	Mobilizing sigmoid colon laterally
15	Mobilizing descending colon laterally
	Mobilizing sigmoid and descending colon laterally
	Mobilizing sigmoid colon laterally
16	Dissecting mesorectum
	Dissecting mesorectum
17	Dissecting descending colon off small bowel mesentery adhesions
18	Dissecting mesorectum
	Dissecting mesorectum
	Dissecting inferior mesentery artery
19	Mobilizing ascending colon laterally
20	Mobilizing sigmoid colon laterally
	Dissecting omentum off distal transverse colon
21	Mobilizing sigmoid colon laterally
22	Mobilizing sigmoid and descending colon laterally
	Dissecting mesorectum
	Dissecting sigmoid colon mesentery medially
	Dissecting mesorectum
	Dissecting mesorectum
	Mobilizing sigmoid colon laterally
	Dissecting inferior mesenteric artery
	Stapling and preparing rectum
23	Dissecting mesorectum
24	Mobilizing ascending colon laterally
	Dissecting ascending colon mesentery

Operating with the presence of VPM for >1-min duration occurred 51 times (5 times with nonergonomic hand positions). The frequency of operating with the presence of VPM was similar during the early and later stages of surgery (Graph S1). Operating with the presence of VPM for >1-min duration involved dissection all 51 times. The target was sigmoid colon in 12 situations, rectum in 9 situations, colon mesentery in 5 situations, ascending colon in 5 situations, splenic flexure in 4 situations, descending colon in 4 situations, hepatic flexure in 4 situations, transverse colon in 4 situations, inferior mesenteric artery in 3 situations, and ileocolic region in 1 situation (Table 2).

## Discussion

The study of the 24 consecutive cases revealed a distinct (>2-fold) preference for the surgeon to maintain his hands in an ergonomic side-by-side forearm-supported position on the console bar, in preference to avoidance of VPM, resulting in up–down misalignment of inside instruments. The potential additional cognitive load of VPM did not affect surgery flow. Although it was an uncommon occurrence, surgery was not impeded even when there was an opposite orientation of the outside surgeon hand and inside instrument positions (true VPM). Operating with the presence of VPM occurred most frequently involved mobilization of the colon from its lateral attachments. This involved retraction of the colon medially and dissection more laterally, resulting in the left-handed instrument tip positioned below the right-handed instrument tip on the screen.

Our study finding contrast with a simulation study, where 45 subjects were tasked with picking up a peg with one robotic arm, passing it to the other arm and then placing it on the opposite side, with and without 10-cm up–down axis mismatch [4]. The misalignment of the robotic controls relative to the robotic arms had a negative impact on task completion. The main difference of this study was the recruitment of novice surgeons and the fact that the misalignment was created at the beginning of the task, which did not allow sufficient time for sensory recalibration that can occur during nonsimulated surgery.

With cross-sensory recalibration, one or both unimodal sensory cues shift unconsciously towards the other in the presence of a mismatch [9]. The surgeon relies on the processes of multisensory integration and sensory recalibration to organize multisensory information into a coherent perception [11]. Addition of haptic feedback has been shown to reduce the negative effects of VPM in a simulation study [4]. The synchronicity of the visual and tactile stimulation may be able to better compensate for the incongruency between the hand/instrument tip positions. In the rubber hand illusion, which involves a spatial visual–proprioceptive mismatch, simultaneous stroking of a subject’s hidden hand and a visible rubber hand induces a transient illusion of the latter feeling like the real hand. Increasing the mismatch between the visual and proprioceptive modality by placing the two hands further apart abolished the illusion [12]. With regards to robotic surgery, brain recalibration allows the surgeon to unconsciously move his/her hands synchronously despite the conflict between the visual and proprioception information as long as the mismatch is not too pronounced.

The resting of the forearms on the robot console bar and the limited confines of the robotic console which restrict large movements of the hands may be other contributing factors that facilitate maintenance of the neutral position. The validated Rapid Upper Limb Assessment tool has confirmed that resting of the forearms results in optimal shoulder posture score [13]. Supporting the weight of the forearm can reduce fatigue and improve the precision of hand movements. The armrest load has been shown to be significantly higher in experts compared with novices during simulated robotic exercises [14]. Clutching of the control manipulators is more common with initial coaching and can help maintain the elbows in a neutral position, avoid shoulder abduction, and reduce tension on the intrinsic hand muscles [15, 16].

There were limitations of our study. Despite analysis of the side-by-side videos by two independent observers, optimal interpretation and complete accuracy could not be guaranteed. We have previously reported that poor upper limb ergonomics with either hand positioned away from the central console position was uncommon in a study which involved concurrent video recordings of the case and surgeon hand position from the back [17]. The displacement of the surgeon hand position in a forwards or backwards direction can be seen on the lateral/sagittal surgeon video recording, but this cannot be appreciated well on the 2D video recording of the case. Only one experienced colorectal surgeon was videotaped in this study, which negatively impacts on the generalizability of the findings. VPM may be a more significant factor for an inexperienced robotic surgeon during their learning phase.

## Conclusion

A previous simulation robotic surgery study involving novice surgeons found that VPM impaired performance. Our study found that VPM was well compensated for during nonsimulated surgery. For an experienced robotic colorectal surgeon, unconscious ergonomic side-by-side positioning of the hands was preferred despite the potential additional cognitive workload of VPM.

## Supplementary Material

graph_1_rjae143
