# Supplementary material for: Ergonomic hand positioning overcomes visual perception mismatch in nonsimulated robotic colorectal surgery
Source: J Surg Case Rep. 2024 Mar 15;2024(3):rjae143. doi: 10.1093/jscr/rjae143 (PMC10941812; doi:10.1093/jscr/rjae143)
Supplement: graph_1_rjae143 [file graph_1_rjae143.docx]

**Graph 1: Occurrence of non-ergonomic position or visual perception mismatch for more than one minute duration in relation to 30-minute operation blocks.**
